# Antifungal Treatment Aggravates Sepsis through the Elimination of Intestinal Fungi

**DOI:** 10.1155/2021/2796700

**Published:** 2021-10-18

**Authors:** Baifa Sheng, Yihui Chen, Lihua Sun, Peng Xu, Ben Han, Xiaolong Li, Jiuheng Yin, Teming Li, Haidi Guan, Shuaishuai Chen, Qi Wang, Chuangen Li, Shiqiang Li, Xianhong Jiang, Peng Wang, Qiuyue He, Yong Wang, Weidong Xiao, Hua Yang

**Affiliations:** ^1^Department of General Surgery, Xinqiao Hospital, Army Medical University, Chongqing 400037, China; ^2^Department of Emergency Medicine, PLA Strategic Support Force Characteristic Medical Center, Beijing 100101, China; ^3^Department of General Surgery, The First Mobile Corps Hospital of PAP, Dingzhou City, 073000 Hebei Province, China; ^4^Department of Nutrition, Xinqiao Hospital, Army Medical University, Chongqing 400037, China; ^5^Division of Hematology-Oncology, Department of Medicine Beth Israel Deaconess Medical Center, Harvard Medical School, Boston, MA 02215, USA; ^6^Department of Laboratory Animal Science, College of Basic Medical Science, Army Medical University, Chongqing 400038, China

## Abstract

Prophylactic antifungal therapy is widely adopted clinically for critical patients and effective in reducing the morbidity of invasive fungal infection and improves outcomes of those diagnosed patients; however, it is not associated with higher overall survival. As intestinal commensal fungi play a fundamental role in the host immune response in health and disease, we propose that antifungal therapy may eliminate intestinal fungi and aggravate another critical syndrome, sepsis. Here, with murine sepsis model, we found that antifungal therapy with fluconazole dismissed intestinal fungal burden and aggravated endotoxin-induced but no gram-positive bacteria-induced sepsis. Nevertheless, antifungal therapy did not exert its detrimental effect on germ-free mice. Moreover, colonizing more commensal fungi in the mouse intestine or administration of fungal cell wall component mannan protected the mice from endotoxin-induced sepsis. On the molecular level, we demonstrated that antifungal therapy aggravated endotoxin sepsis through promoting Gasdermin D cleavage in the distal small intestine. Intestinal colonization with commensal fungi inhibited Gasdermin D cleavage in response to lipopolysaccharide challenge. These findings show that intestinal fungi inhibit Gasdermin D-mediated pyroptosis and protect the mice from endotoxin-induced sepsis. This study demonstrates the protective role of intestinal fungi in the pathogenesis of endotoxin-induced sepsis in the laboratory. It will undoubtedly prompt us to study the relationship between antifungal therapy and sepsis in critical patients who are susceptible to endotoxin-induced sepsis in the future.

## 1. Introduction

Invasive fungal infections (IFIs) are life-threatening for critical patients and have a mortality rate of approximately 40-55% in intensive care units (ICUs) [1–3]. Timely adoption of antifungal therapy is crucial for improving the outcomes of definite IFI patients [4]; thus, prophylactic or empirical antifungal treatment (AFT) for those without definite IFI is attractive. Despite the fact that the prophylactic AFT is widely adopted in daily clinical practice for those at high risk of IFI such as patients with hematological malignancies, those who underwent gastrointestinal surgery, or premature infants [5–9], divergences exist all the time [10–13]. One focus is whether or not prophylactic AFT improves the overall survival of patients. An increasing number of clinical studies and systemic reviews have concluded that prophylactic AFT significantly reduces IFI morbidity and IFI-associated mortality but is not associated with improvement in overall survival [12, 14, 15]. This indicates that AFT may increase the morbidity or mortality of other severe conditions in the ICU; however, this proposal has not been validated clinically or in a laboratory until now. Since sepsis is another severe syndrome in the ICU, we propose that prophylactic AFT aggravates sepsis.

Sepsis is defined as life-threatening organ dysfunction caused by a dysregulated host response to infection and is one of the leading causes of deaths in ICUs [16]. Acute inflammation plays a defensive role in eliminating invading pathogens by producing proinflammatory cytokines. However, sometimes, the pathogen may persist, and the host response becomes uncontrolled, resulting in organ damage as seen in sepsis [17]. The detrimental immune response underlying sepsis is elicited by innate immune cells sensing pathogen-associated molecular patterns (PAMPs) with their pattern recognition receptors (PRRs) [18]. Lipopolysaccharide (LPS), a gram-negative bacterial cell wall component, is a typical PAMP. When high dose of LPS is recognized by host's PRRs, it results in sepsis/septic shock in mice [19]. Recent studies have revealed the determinant role of Gasdermin D- (GSDMD-) mediated pyroptosis in the pathogenesis of sepsis [20, 21]. Cleaved by the proteinase cysteine-dependent aspartate proteases (CASP) 1/11 in mice or by CASP4/5 in humans, the GSDMD N-terminal P30 fragment forms pores on the cell membrane, ultimately resulting in cell death, pyroptosis. Dead cells, especially macrophages or monocytes, release proinflammatory cytokines IL-1*β*, IL-18, or other damage-associated molecular patterns. As a result, inflammatory and coagulation cascades initiate and induce septic shock or death in mice [22, 23]. GSDMD is essential for LPS-induced lethal sepsis, which verifies the central role of GSDMD-mediated pyroptosis in determining sepsis outcome [23–26].

The mammalian intestine harbors trillions of microbes, including commensal fungi, which play an indispensable role in health and disease [27]. Fungal dysbiosis resulted from antifungal drug administration or fungal receptor gene mutation leads to susceptibility to local colitis, colon cancer, or remote organ lung allergic disease in mouse models or patients [28–30]. Clinically, AFT is widely used to treat definite fungal infections or to prevent possible IFIs for critical patients. This will undoubtedly lead to fungal dysbiosis and may have unknown consequences on sepsis pathogenesis.

In this study, we found the AFT dismissed intestinal fungal burden and decreased the survival rate and aggravated organ injury in gram-negative bacteria or LPS-induced sepsis. Colonization with a single fungal strain or supplementing the fungal cell wall component, mannan, in drinking water could improve the sepsis outcome. At the molecular level, AFT increases GSDMD cleavage in the distal small intestine, while commensal intestinal fungi inhibit GSDMD cleavage in response to LPS. Additionally, AFT did not exert detrimental effect on GSDMD-deficient mice. This study provides a new perspective on prophylactic AFT and bacterial sepsis, and further laboratory and clinical studies are needed to further this research.

## 2. Materials and Methods

### 2.1. Animals

6- to 10-week-old C57BL/6J mice were used in this study. All the wild-type mice were purchased from Gempharmatech (Nanjing, Jiangsu, China) and maintained under specific pathogen-free (SPF) condition at the animal center of Xinqiao Hospital, Army Medical University. The Germ-free C57BL/6J mice were maintained in the Medical Animal Center of Army Medical University. GSDMD-deficient mice (*Gsdmd^−/−^*) were kindly gifted by Dr. Shu Zhu from the University of Science and Technology of China. All operations and treatments to the mice were performed in accordance with the guidelines of the Institutional Animal Care and Use Committee of the Army Medical University.

### 2.2. Drugs Treatment

Fluconazole (Sigma, St. Louis, MO, USA) or amphotericin B (Sango biotech, Shanghai, China) was dissolved at concentration of 0.5 mg/mL and 0.1 mg/mL, respectively, in mouse drinking water and changed every 3 days to refresh the reagents. To treat hamsters, 5 mg/mL fluconazole was given orally at 100 *μ*L/day/hamster. Antibiotics (Abx) were made by dissolving four antibiotics (Sango Biotech, Shanghai, China), namely, ampicillin (10 mg/mL), neomycin (10 mg/mL), vancomycin (5 mg/mL), and metronidazole (10 mg/mL), in their drinking water, and 200 *μ*L of this drinking water was given per mouse daily by gavage. Disulfiram (Sigma, St. Louis, MO, USA) was formulated in olive oil at the concentration of 5 mg/mL and injected intraperitoneally at a dose of 50 mg/kg 24 hours and 4 hours before LPS challenge.

### 2.3. Sepsis Model

The endotoxic shock mouse model was constructed via intraperitoneal injection of 10 mg/kg LPS (Sigma, St. Louis, MO, USA). The cecal ligation and puncture (CLP) sepsis model was induced as described in the literature with minor modifications [31]. Briefly, 6- to 10-week-old age and sex-matched mice were anesthetized with 1% entobarbital sodium (10 mL/kg) intraperitoneally, and a 1 cm incision was made on their abdomen. The exposed cecum was ligated 0.5 cm from the cecal end, and a single through and through puncture was performed. The cecum was placed back into the peritoneal cavity, and the abdomen was closed. Finally, 1 mL of warm normal saline was injected intraperitoneally. To establish the methicillin-resistant *Staphylococcus aureus* (MRSA) sepsis model, 1 × 10^8^ colony-forming unit (CFU) of the bacteria was injected intraperitoneally. 1 × 10^4^ CFU *Salmonella typhimurine* SL1344 was injected intraperitoneally to induce gram-negative bacterial sepsis.

### 2.4. Blood Sampling

Ether-anesthetized mice blood were collected from retro-orbital plexus using a capillary tube. The blood samples were coagulated in room temperature for 1 hour and then centrifuged for serum at 12,000 g for 10 minutes at 4°C.

### 2.5. LEGENDplex™ Assays

Serum cytokine levels were determined using the LEGENDplex™ assay kit (BioLegend, San Diego, CA, USA) according to manufacturer's instructions. Samples were run on Gallios (Beckman Coulter, Brea, CA, USA), and data were analyzed online (https://legendplex.qognit.com/workflow).

### 2.6. Histology

Paraffin-embedded tissue sections were cut into 5 *μ*m thick sections and stained with hematoxylin and eosin. Images were taken using a fluorescence microscope (IX71, Olympus, Tokyo, Japan) under a bright field.

### 2.7. Fecal DNA Isolation and Fungal and Bacterial rDNA Quantitative Analysis

Weighed fecal pellets were beaded with 250 *μ*L 20% SDS, and 500 *μ*L PB buffer (Qiagen, Hilden, NRW, Germany), 200 *μ*L 0.1 mm beads (Biospec, USA), 550 *μ*L phenol : chloroform : IAA (Solabio, Beijing, China). After centrifuging at 8,000 rpm for 3 minutes at room temperature, the aqueous phase was collected and purified using the Qiagen PCR purification kit (Qiagen, Hilden, Germany). One hundred nanograms fecal DNA was used as a template for quantitative PCR analysis, which was performed on the CFX96 real-time system (Bio-Rad, Hercules, CA, USA) with the SYBR mix of GoTaq® PCR Master Mix (Promega, Madison, WI, USA). Total fungal burden was calculated using the △Ct method and normalized to the total fecal DNA content and weight of fecal samples. The primer sequences used were as follows:

18S-FAGGCGCGCAAATTACCCAATCC

18S-RGCCCTCCAATTGTTCCTCGTTAAG

ITS2-FCTTGGTCATTTAGAGGAAGTAA

ITS2-RGCTGCGTTCTTCATCGATGC

*C. albicans*-FCTGTTTGAGCGTCGTTTC

*C. albicans*-RATGCTTAAGTTCAGCGGGTAG

*C. tropicalis*-FTTTGGTGGCGGGAGCAATCCT

*C. tropicalis*-RCGATGCGAGAACCAAGAGATCCGT

### 2.8. Fungal Monocolonization and Mannan Administration

Single *Candida tropicalis* ATCC 13803 or *Candida albicans* CMCC (F) 98001 clone was picked and shaked in yeast extract peptone dextrose medium (Solabio, Beijing, China) at 30°C in incubator overnight. The next day, 5 × 10^7^ CFU of fungi were administrated by oral gavage to the indicated group. Mannan (Sigma, St. Louis, MO, USA) was dissolved in mouse drinking water at a concentration of 0.1 mg/mL.

### 2.9. Western Blot

Mouse ileum samples were harvested from the small intestine at a distance of 5 cm from the cecum and then homogenated in ice-cold radioimmunoprecipitation assay buffer (Solabio, Beijing, China) supplemented with 1% phenylmethanesulfonyl fluoride (Boster, Wuhan, China). The homogenate was incubated on ice for 30 minutes and then centrifuged at 12,000 g for 10 minutes at 4°C. The tissue protein extract was separated by sodium dodecyl sulphate-polyacrylamide gel electrophoresis and transferred to a polyvinylidene fluoride membrane. Western blot analysis was performed with GSDMD (Abcam, Cambridge, MA, USA) or glyceraldehyde 3-phosphate dehydrogenase (Cell signaling Technology, Beverly, MA, USA) antibodies.

### 2.10. Serum ALT (Alanine Aminotransferase) Measurement

Serum ALT was measured using an alanine aminotransferase assay kit (Jiancheng, Nanjing, China) according to manufacturer's instructions.

### 2.11. Statistical Analysis

The measurement data are expressed as the mean ± SD. Two-group comparisons were conducted with a standard two-tailed unpaired Student's *t*-test, and multiple comparisons were performed using one-way analysis of variance followed by Tukey's multiple comparisons test. Mortality was compared using Kaplan-Meier survival curves and analyzed using the log-rank (Mantel-Cox) test. All calculations were performed using GraphPad Prism (Madison, WI, USA). ^∗^*p* < 0.05, ^∗∗^*p* < 0.01, ^∗∗∗^*p* < 0.001, ^∗∗∗∗^*p* < 0.0001 unless indicated.

## 3. Results

### 3.1. AFT Aggravates Murine Sepsis Model

**Figure 1:**
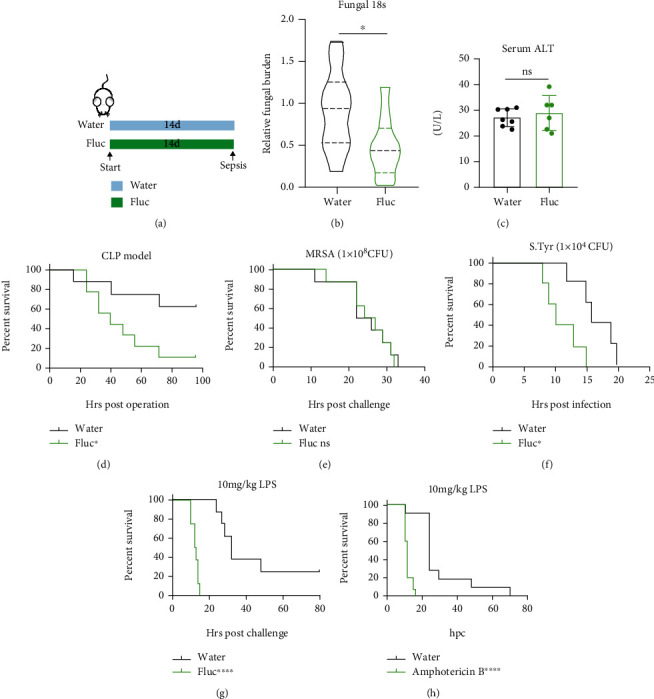
Antifungal treatment hastened death of mouse sepsis model. (a) Layout of the experimental setup; Fluc represents fluconazole (b–g). (b) Relative fungal burden in mouse stool was determined by qPCR of fungal 18 s rDNA, *n* = 9-10 per group. (c) Serum alanine aminotransferase (ALT) level at the 14^th^ day of indicated groups, *n* = 6-7 per group. (d) Survival curve of the cecal ligation and puncture (CLP) model, *n* = 8-9 per group. (e) Survival curve of the MRSA-induced sepsis model at the dose of 1 × 10^8^ CFU/mouse, *n* = 8 per group. (f) Survival curve of the *Salmonella typhimurium*- (S.Tyr-) induced sepsis model at the dose of 1 × 10^5^ CFU/mouse, *n* = 5 per group. (g) Survival curve of the lipopolysaccharide- (LPS-) induced sepsis model, *n* = 10 per group. (h) Survival curve of the LPS-induced sepsis model on the water control or amphotericin B treated mice, *n* = 10 per group. Data were from one of two (b–e) or four independent experiments (g). Two group comparisons were conducted with a standard two-tailed unpaired Student's *t*-test (b, c). Mortality was compared by Kaplan-Meier survival curves and analyzed by the log-rank (Mantel-Cox) test (d–h).

To explore the effects of prophylactic AFT on the pathogenesis of murine sepsis, we pretreated C57/B6 mice with fluconazole in their drinking water at a concentration of 0.5 mg/mL for two weeks and then performed the CLP sepsis procedure (Figure 1(a)). Although fluconazole reduced the fungal burden in mouse stool (Figure 1(b)) and had no definite toxicity in the liver (Figure 1(c)), the survival rate of the fluconazole-treated group was unexpectedly dramatically lower than that of the control group (Figure 1(d)). As CLP causes sepsis of polymicrobial infection, we wondered whether gram-positive or gram-negative bacteria-induced sepsis was aggravated by fluconazole treatment. When sepsis was induced with the gram-positive bacteria MRSA, fluconazole pretreatment had no effects on the survival (Figure 1(e)). On the contrary, fluconazole pretreatment worsened the gram-negative bacteria *Salmonella typhimurine*-induced sepsis (Figure 1(f)). This led us to speculate that the pathogenic factors, specifically owned by the gram-negative bacteria that triggers sepsis, were affected by AFT. As LPS is the prominent effector that triggers sepsis in gram-negative bacteria, we tested and confirmed that AFT aggravates endotoxin-induced sepsis (Figure 1(g)). To determine whether other antifungal drugs had similar effects, we pretreated mice with another antimycotic, amphotericin B, which also exerted detrimental effects on LPS-induced sepsis (Figure 1(h)).

**Figure 2:**
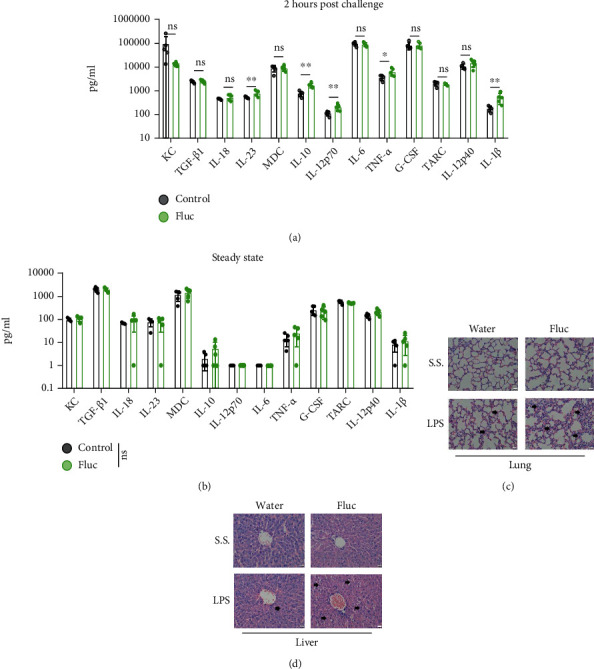
Antifungal treatment augmented cytokine production and aggravated organ injury. (a, b) Serum cytokine levels were determined by LEGENDplex™ assays 2 hours post lipopolysaccharide (LPS) challenge (a) or before LPS challenge (b), *n* = 5 per group. (c, d) Representative fields of view from hematoxylin and eosin-stained sections showed the lung (c) and liver (d) tissue of mice at the 14^th^ day of fluconazole treatment versus water treatment control on steady state (S.S.) or 10 hours post-LPS challenge. The black arrow indicates the tissue damage or immune cell infiltration. Scale bar represents 20 *μ*m. Two group comparisons were conducted with a standard two-tailed unpaired Student's *t*-test (a, b).

To evaluate which inflammatory responses were amplified by AFT, we collected mouse serum before LPS injection or at early time points (2 hours) after the LPS challenge and measured cytokine levels with a cytokine panel. In accordance with the survival rate, the fluconazole-treated group produced more proinflammatory cytokines (IL-1*β*, TNF-*α*, IL-23, and IL-12p70) and the anti-inflammatory cytokine IL-10 upon LPS challenge (Figure 2(a)) but not at steady state (Figure 2(b)). In addition, tissue biopsies of the lung (Figure 2(c)) or liver (Figure 2(d)) at 10 hours post-LPS challenge also showed severe tissue damage and increased immune cell infiltration in the fluconazole-treated group; this time point was widely adopted to evaluate tissue damage in endotoxic shock model [32, 33]. In summary, AFT predisposed mice to lethal sepsis/endotoxin shock.

### 3.2. AFT Aggravates Endotoxin-Induced Sepsis via Fungal Microbiota

**Figure 3:**
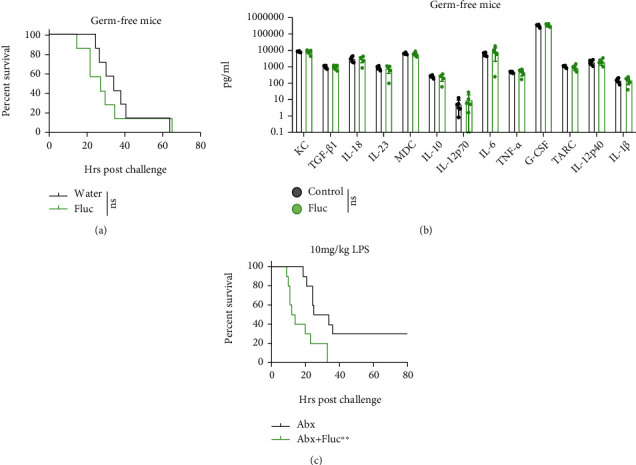
Antifungal treatment aggravated sepsis model via fungal microbiota. (a) Survival curve of germ-free mice in water drinking (control) or fluconazole drinking group in challenge of lipopolysaccharide (LPS), *n* = 7 per group. (b) Serum level of cytokines of germ-free mice at 10 hours post-LPS challenge of the two groups, *n* = 6 per group. (c) Survival curve of antibiotics- (abx-) treated and abx plus fluconazole treated mice. *n* = 10 per group. Data were from one of two independent experiments (c). Two group comparisons were conducted with a standard two-tailed unpaired Student's *t*-test (b). Mortality was compared by Kaplan-Meier survival curves and analyzed by the log-rank (Mantel-Cox) test (a, c).

Because the intestinal microbiota, especially the intestinal fungi, which play a fundamental role in health and disease, would be disturbed by administration of the broad-spectrum antifungal drugs fluconazole or amphotericin B, we treated germ-free mice with fluconazole to verify whether ATF aggravates sepsis by directly acting on the host or indirectly by modulating the microbiota. According to our data, AFT did not hasten the death of the LPS-induced sepsis model (Figure 3(a)). Moreover, the serum cytokine levels did not show any difference on challenge of LPS (Figure 3(b)), which indicated a comparable immune response between the two groups in the background of germ-free mice. To further investigate whether the fungal microbiota mediate the AFT effect, we pretreated mice with Abx to clear intestinal bacteria, after which AFT still aggravated the LPS-induced sepsis model (Figure 3(c)). These results indicate that it is most likely that the elimination of intestinal fungi confers the detrimental effect of AFT in LPS-induced sepsis.

### 3.3. Intestinal Fungi Protect Mice from LPS-Induced Sepsis

**Figure 4:**
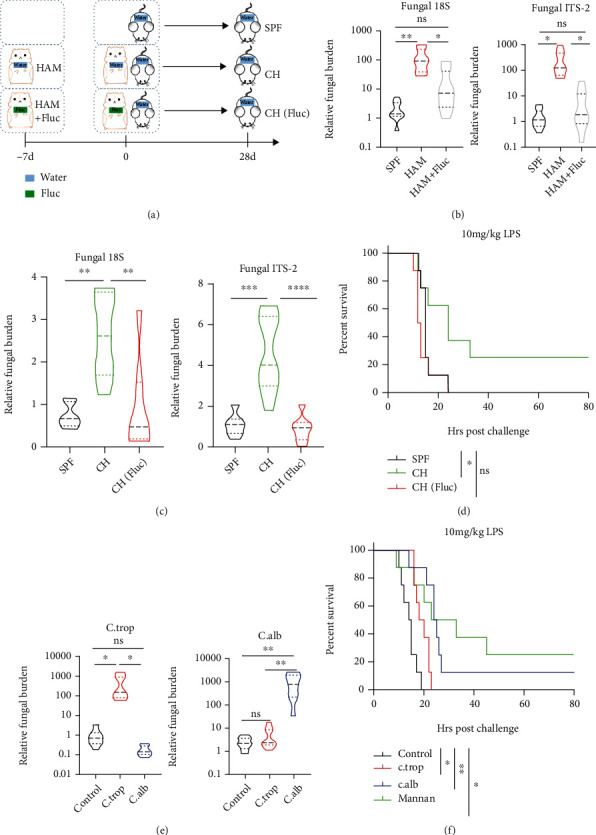
Intestinal fungi protected mice from lipopolysaccharide- (LPS-) induced sepsis. (a) Layout of the cohousing (CH) experiment setup (b–d). Wild hamsters purchased from the pet market were pretreated with water (HAM) or fluconazole (HAM+Fluc) for 7 days first. At day 0, specific pathogen-free (SPF) mice were isolated housed (SPF group) or cohoused with the water-treated (CH group) or Fluc-treated (CH+Fluc group) hamsters. Twenty-eight days later, C57B6 mice were challenged with LPS for modeling. (b) At day 0, relative fungal burden in SPF, HAM, and HAM+Fluc groups was measured with fungal 18S, ITS-2 ribosomal DNA relative copies in total fecal DNA, *n* = 8 per group. (c) At day 28, relative fecal fungal burden were measured in SPF, CH, and CH (Fluc) groups, *n* = 7 per group. (d) Survival rate of the different groups of C57B6 mice as shown in (a) in challenge with LPS, *n* = 8 per group. (e) Relative fecal DNA copies of *Candida tropicalis* (C.trop) or *Candida albicans* (C.alb) in SPF mice (control group), C.trop colonized mice and C.alb colonized mice one week after colonization, *n* = 8 per group. (f) Survival rate of the indicated groups in challenge with LPS. LPS was injected at the 30^th^ day of fungal strain colonization or mannan administration, *n* = 8 per group. Multiple comparisons were performed with one-way analysis of variance followed by Tukey's multiple comparisons test (b, c, e). Mortality was compared by Kaplan-Meier survival curves and analyzed by the log-rank (Mantel-Cox) test (d, f). Adjusted *p* < 0.025 for ^∗^ (d), *p* < 0.017 for ^∗^, and *p* < 0.003 for ^∗∗^ (f).

Since AFT dismissed fecal fungal burden and predisposed mice susceptible to LPS-induced lethal sepsis, we assumed that the commensal fungi that preexisted in the intestinal tract might be essential in restraining the sepsis process. To test our hypothesis, we performed a cohousing experiment by breeding SPF C57/B6 mice with wild hamsters at ration of 1 : 1 for 4 weeks (Figure 4(a)), which would balance their microbiota, including the intestinal fungi. This experiment has two advantages: first, the wild hamster would transfer more commensal fungi to the C57/B6 mice in a natural way; second, this could avoid the possible direct toxicity that fluconazole has on SPF C57/B6 mice. By quantifying the relative fungal DNA copies in the feces, we found that the hamsters were colonized with more abundant fungi in their intestine and treating hamsters with fluconazole for 7 days dismissed the fungal burden equal to our SPF mice, as shown by the relative DNA copies of fungal 18S and internal transcribed spacer 2 (ITS-2) in the feces (Figure 4(b)). After 4 weeks of cohousing, the C57/B6 mice gained more intestinal fungi than the isolated housed mice. In contrast, C57/B6 mice that were cohoused with fluconazole-treated hamster maintained low fungal burden equal to the SPF mice, as evidenced by the fecal fungal 18s and ITS-2 quantification (Figure 4(c)). As a result, the mice that gained wild hamster intestinal fungi showed resistance to LPS-induced sepsis compared to the SPF mice. On the other hand, the mice cohoused with fluconazole-treated hamsters did not acquire the protection (Figure 4(d)). To validate whether a single intestinal commensal fungal strain could recapitulate the protective effect, we colonized singly using the fungal strain *Candida tropicalis* and *Candida albicans*, which are prevalent in mouse or human intestine, respectively [28, 34], by oral gavage (Figure 4(e)) or administered the major fungal cell wall component mannan in the mouse drinking water for 1 month before sepsis modeling. It was observed that the treated mice were resistant to the endotoxin-induced sepsis as compared with the control mice (Figure 4(f)). These results demonstrate that the whole wild intestinal fungi, a single commensal fungal strain, or even just the fungal surface component mannan, could confer protection against LPS-induced sepsis.

### 3.4. Intestinal Fungi Protected Mice from LPS-Induced Sepsis through Inhibiting GSDMD Cleavage

**Figure 5:**
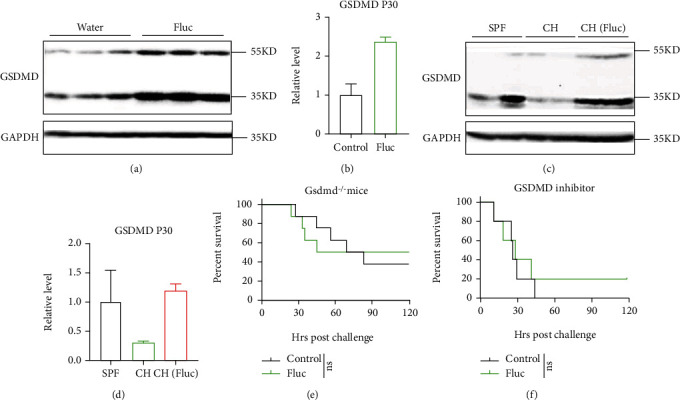
Intestinal fungi protected mice from LPS-induced sepsis through inhibiting GSDMD cleavage. (a) GSDMD cleavage of ileal tissue in water control and fluconazole-treated group 2 hours post-LPS challenge. The 53 KD and 30 KD represent the full length and N-terminal fragment of GSDMD, respectively. (b) Relative protein level of GSDMD P30 measured with Image J of (a). (c) GSDMD cleavage of ileal tissue in the SPF mice grouped as described in Figure 4(a) 2 hours post-LPS challenge. (d) Relative protein level of GSDMD P30 measured with Image J of (c). (e) Survival rate of LPS-induced sepsis in indicated groups. The *Gsdmd*-deficient (*Gsdmd^−/−^*) mice were treated with water (control) or fluconazole (Fluc) for two weeks before LPS challenge. *n* = 8 for each group. (f) The survival rate of LPS-induced sepsis in indicated groups. The GSDMD inhibitor disulfiram was administrated at the 14^th^ day of the water or fluconazole treated mice. *n* = 5 per group. Mortality was compared by Kaplan-Meier survival curves and analyzed by the log-rank (Mantel-Cox) test (e, f).

As the cell death form pyroptosis downstream of GSDMD cleavage plays a determinant role in LPS-induced sepsis and the fact that the ileum is the intersection of endotoxin shock and fungal colonization [28, 33, 35], we detected GSDMD protein level in the ileal tissue and observed that AFT increased GSDMD cleavage in the ileum upon challenge with LPS (Figures 5(a) and 5(b)). In consistence with this, transferring intestinal fungi to the SPF mice by cohousing them with wild hamsters inhibited GSDMD cleavage in the ileum, and pretreating hamsters with fluconazole did not transfer this inhibitory effect at 2 hours post-LPS injection (Figures 5(c) and 5(d)). Finally, in order to prove that AFT accelerated mouse death by increasing GSDMD-mediated pyroptosis, we treated *Gsdmd^−/−^* mice with fluconazole or applied a GSDMD inhibitor, disulfiram, which could inhibit pore formation on cell membrane by GSDMD N-terminal fragment and thus halt pyroptosis-mediated sepsis progressing [35]. The results showed that *Gsdmd* gene deletion (Figure 5(e)) or functional blockage of GSDMD (Figure 5(f)) could completely abolish the detrimental effect of AFT. These results indicate that intestinal fungi protect mice from LPS-induced sepsis by inhibiting GSDMD-mediated pyroptosis.

## 4. Discussion

Here, we demonstrated that AFT with fluconazole or amphotericin B aggravates endotoxin-induced sepsis laboratorially. The dose or the method of the fluconazole administration has been widely used in previous mouse experiments [28, 36–38]. This gave rise to a serum fluconazole concentration of approximately 20 *μ*M [29], which is lower than the human serum concentration of about 20 mg/L (65 *μ*M) by administrating 400 mg fluconazole every 24 hours [39]. This implies that the detrimental effect of AFT observed here can probably be applied to human patients.

Although AFT is widely adopted clinically, few studies have attempted to understand the relationship between AFT and bacterial sepsis as most studies have only focused on AFT and fungi-associated sepsis. A prospective study showed that fluconazole use improved survival in patients with septic shock [40]. It should be noted that the patients enrolled in this study were already diagnosed with septic shock before fluconazole administration, and thus, the inflammatory cascades have already been initiated. Fluconazole may improve survival by inhibiting the intestinal opportunistic fungal pathogens that would cause IFI in immunocompromised septic shock patients [2]. Furthermore, this study did not distinguish the pathogen of septic shock, as our results showed that only endotoxin shock was worsened by AFT. Another point is that this was a solitary report from only one center. More clinical studies are needed to understand and verify the relationship between AFT and bacterial sepsis. In addition to the scarce clinical studies, few laboratory studies have attempted to explore this topic.

In the 2000s, a group reported the relationship between fluconazole treatment and peritonitis or sepsis model [41, 42]. However, they drew the opposite conclusion that fluconazole treatment attenuates lung injury and mortality in a fecal peritonitis sepsis model. Nevertheless, attention should be paid on certain aspects of the study. First, the way in which fluconazole was used in this study is quite different from ours. The authors administered fluconazole orally just 30 minutes before fecal inoculation. We found that pretreating mice with fluconazole for 14 days would worsen their sepsis. Clinically, the prophylactic use of AFT ranges from days to months depending on the clinical setting [7, 9, 43, 44]. Therefore, our design better mimics the prophylactic administration of AFT. Second, unlike the 30 minutes pretreatment done in their study, 14 days AFT could have a great impact on the mycobiome of the intestine, eventually exerting adverse effects on the murine disease model [27, 45]. Until now, there is limited evidence showing what role the preexisting commensal fungi plays in the pathogenesis of sepsis. Although the intestinal mycobiome has been demonstrated to play an important role in the pathogenesis of colitis, colorectal cancer, allergic airway disease, and influenza A virus infection [28, 29, 37, 46]. In our study, we showed that the intestinal commensal fungal colonization inhibited GSDMD cleavage in the ileum and protected mice from endotoxin shock.

The finding that commensal fungi colonization inhibits GSDMD cleavage in response to LPS challenge prompted us to question whether the commensal fungi directly inhibit the GSDMD cleavage or if it is just the result of the mucosal immune system adapting to the fungal colonization. We prefer the latter. Orally, administration of *Candida albicans* for 3 hours or 5 days was reported to worsen CLP sepsis model [47, 48]. However, we find intestinal colonization of *Candida albicans* for 1 month protects mice from endotoxin-induced sepsis. So, we assume that the host adaptation to the fungi colonization restrains sepsis response; this adaptation will take a certain period of time. In support of our notion, another study reported 14 days colonization with *Candida albicans*-protected mice from influenza A virus intranasal infection and dextran sodium sulfate induce colitis [37]. As to which cell type or molecule that mediate the adaptation requires more detailed research in the future. It is known that GSDMD cleavage in response to LPS challenge is downstream of the noncanonical inflammasome pathway [49]. However, whether upstream components of GSDMD, such as CASP11 signaling, are regulated by commensal fungi colonization, also needs to be explored.

Although the experimental evidence clearly shows that fluconazole or amphotericin B pretreatment worsens sepsis outcome by eliminating intestinal fungi and promoting GSDMD cleavage, it is prudent to translate it to the clinics. The reason for this is the complex pathogenesis of sepsis. As mentioned above, the inflammatory reaction is a double-edged sword, and GSDMD-mediated pyroptosis is the mechanism by which the host defends against gram-negative bacteria [50, 51]; however, it is also the key step in the endotoxin shock pathogenesis [35, 52]. It is the battle between the host and pathogen which determines the final outcome. Systemic infection of mice with series doses of gram-negative bacteria may help to dissect the role of AFT in antibacterial response or sepsis.

## 5. Conclusion

We found that AFT with fluconazole dismisses the intestinal fungal burden and predisposes the mice susceptible to lethal septic shock by promoting GSDMD cleavage. We also found that intestinal colonization with more commensal fungi reverses this progression and protects mice from LPS-induced sepsis. This opens a new prospective on antifungal therapy for critical patients with high risk of developing endotoxin sepsis and will probably help to reevaluate the importance of preserving intact fungi in restraining sepsis pathogenesis. However, there is still a long way to elucidate the relationship between AFT and sepsis both clinically and in the laboratory.

## Data Availability

Raw data supporting conclusions in this article are available upon request from the corresponding authors.
